# Near‐Infrared Afterglow Imaging‐Guided Surgical Resection and Synergistic Photodynamic‐Chemo Therapy of Breast Cancer

**DOI:** 10.1002/advs.202503883

**Published:** 2025-06-10

**Authors:** Zixuan Li, Ranran Zhao, Qing Pei, Zhigang Xie, Min Zheng

**Affiliations:** ^1^ School of Chemistry and Life Science Advanced Institute of Materials Science Changchun University of Technology 2055 Yanan Street Changchun Jilin 130012 P. R. China; ^2^ Key Laboratory of Polymer Ecomaterials Changchun Institute of Applied Chemistry Chinese Academy of Sciences 5625 Renmin Street Changchun Jilin 130022 P. R. China

**Keywords:** afterglow, carbon dots (CDs), imaging, photodynamic‐chemotherapy, surgical resection

## Abstract

The recurrence rate of cancer following surgical procedures is markedly affected by the degree of tumor removal. During the operation, tumor‐targeted imaging is of crucial significance as it assists surgeons in attaining the most comprehensive tumor excision. Herein, a theranostic platform (CDSP NPs) is developed through the assembly of carbon dots (CDs) with paclitaxel prodrugs. CDSP NPs can be utilized for near‐infrared (NIR) afterglow imaging‐guided precise removal of breast cancer, featuring a long lifetime (>2 h), deep tissue penetration (>13 mm), and a high signal‐to‐noise ratio (SNR, 103.9), thereby effectively preventing cancer recurrence. Additionally, the combined treatment of photodynamic therapy (PDT) and chemotherapy substantially enhances tumor regression, demonstrating the tremendous potential of CDSP NPs for synergistic cancer treatment. This study proposes a straightforward but efficient model to build a nanoplatform integrating diagnosis and treatment, which is utilized for image‐guided surgical navigation and effective tumor treatment.

## Graphical abstract

A CDSP NPs theranostic platform assembled from CDs and paclitaxel prodrugs is developed. CDSP NPs have long‐lasting NIR afterglow, penetrate deep tissues, and offer a high signal‐to‐noise ratio, enabling NIR afterglow imaging‐guided precise breast cancer removal to prevent recurrence. Moreover, the combination of photodynamic therapy with chemotherapy significantly improves tumor regression, highlighting CDSP NPs' potential for synergistic cancer treatment.

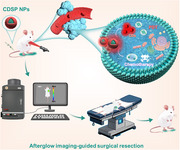

## 1 Introduction

Breast cancer ranks among the prevalent malignant tumors afflicting women.^[^ ^1^ ^]^ At present, surgical excision stands as the favored therapeutic approach for breast cancer in its early stage. However, as many breast tumors are nonpalpable, the existence of residual tumor tissue after surgery can still lead to cancer recurrence and progression. Consequently, achieving the complete elimination of tumor tissues presents a significant challenge. To enhance the efficacy of surgical procedures and improve survival prospects, a novel perioperative treatment strategy utilizing multifunctional platforms has been developed.^[^ ^2^ ^]^ This approach during surgical excision assists in managing micrometastasis and reduces the challenges and risks associated with surgery.^[^ ^3^ ^]^ Within this framework, theranostic nanoplatforms, which integrate diagnostic and therapeutic capabilities, have attracted significant attention as a cutting‐edge development in targeted therapeutic interventions.

Tumor specific imaging before and during surgery is of vital importance in guiding surgeons to achieve the greatest extent of tumor removal.^[^ ^4^ ^]^ Conventional diagnostic imaging modalities like magnetic resonance imaging and computed tomography are mainly utilized for preoperative planning instead of intraoperative guidance. These approaches fall short when it comes to localizing tumors and defining a safe margin for tumors that are not discernible to the naked eye during surgery. Consequently, there is an immediate need to devise novel and dependable strategies that enable precise tumor identification in intricate surgical operations. Recently, optical imaging has gained prominence in intraoperative navigation due to its distinct advantages, which include real‐time visualization, immediate feedback, ease of use, cost‐effectiveness, and safety.^[^ ^5^ ^]^ However, conventional fluorescence imaging techniques that rely on continuous external light excitation are susceptible to interference from tissue autofluorescence.^[^ ^6^ ^]^ In contrast, afterglow imaging with long‐wavelength emission has a superior signal‐to‐noise ratio (SNR) and greater tissue penetration depth as it is not affected by excitation light or tissue interference.^[^ ^7^ ^]^ Therefore, afterglow imaging can enhance the precision of tumor excision and decrease the probability of residual tumor tissue,^[^ ^8^ ^]^ thereby aiding in the management of micrometastasis and reducing the risk of cancer recurrence after surgery.

Chemotherapy serves as a fundamental pillar of drug‐based treatments in the clinical management of breast cancer.^[^ ^9^ ^]^ However, the difficulties connected with chemotherapeutic drugs lie in their weak water solubility and the presence of systemic toxicity, both of which contribute to a reduced therapeutic index.^[^ ^10^ ^]^ Studies have validated that combination therapy can significantly enhance the therapeutic effectiveness of individual chemotherapeutic drugs.^[^ ^11^ ^]^ Photodynamic Therapy (PDT) employs photosensitizers (PSs) that are activated by light of a specific wavelength. This activation then leads to the generation of a significant amount of reactive oxygen species (ROS), which has the effect of eliminating cancer cells.^[^ ^12^ ^]^ PDT presents merits like low invasiveness, strong target selectivity, and a small likelihood of resistance occurrence. Nevertheless, several issues impede the application of PDT, such as the limited penetration depth and the hypoxic tumor microenvironment.^[^ ^13^ ^]^ To improve the therapeutic outcomes for breast cancer, the integration of PDT with chemotherapy represents a promising strategic approach.^[^ ^14^ ^]^ Moreover, imaging‐guided combination therapy has been proven to achieve optimal synergistic effects.^[^ ^15^ ^]^

Carbon dots (CDs), which are newly emerged carbon‐based luminescent nanomaterials, exhibit outstanding optical characteristics, possess a low level of biotoxicity, and are easy to prepare,^[^ ^16^ ^]^ which have spurred extensive research in fields such as biological imaging^[^ ^17^ ^]^ and cancer therapy.^[^ ^18^ ^]^ Up to the present, some CDs‐based phosphors have been fabricated by embedding CDs into organic or inorganic matrices.^[^ ^19^ ^]^ Nevertheless, the majority of them are water‐insoluble and/or their luminescence is severely quenched in an aqueous solution.^[^ ^20^ ^]^ Additionally, phosphorescence typically emits in the short wavelength region,^[^ ^21^ ^]^ and CDs with NIR afterglow are still scarce.^[^ ^22^ ^]^ In our previous work, water‐soluble NIR luminescent CDs were engineered for afterglow imaging‐guided PDT.^[^ ^23^ ^]^ However, hitherto, a theranostic platform that combines imaging‐guided surgical procedures with drug delivery modalities for breast cancer therapy has yet to be established.

**Scheme 1:**
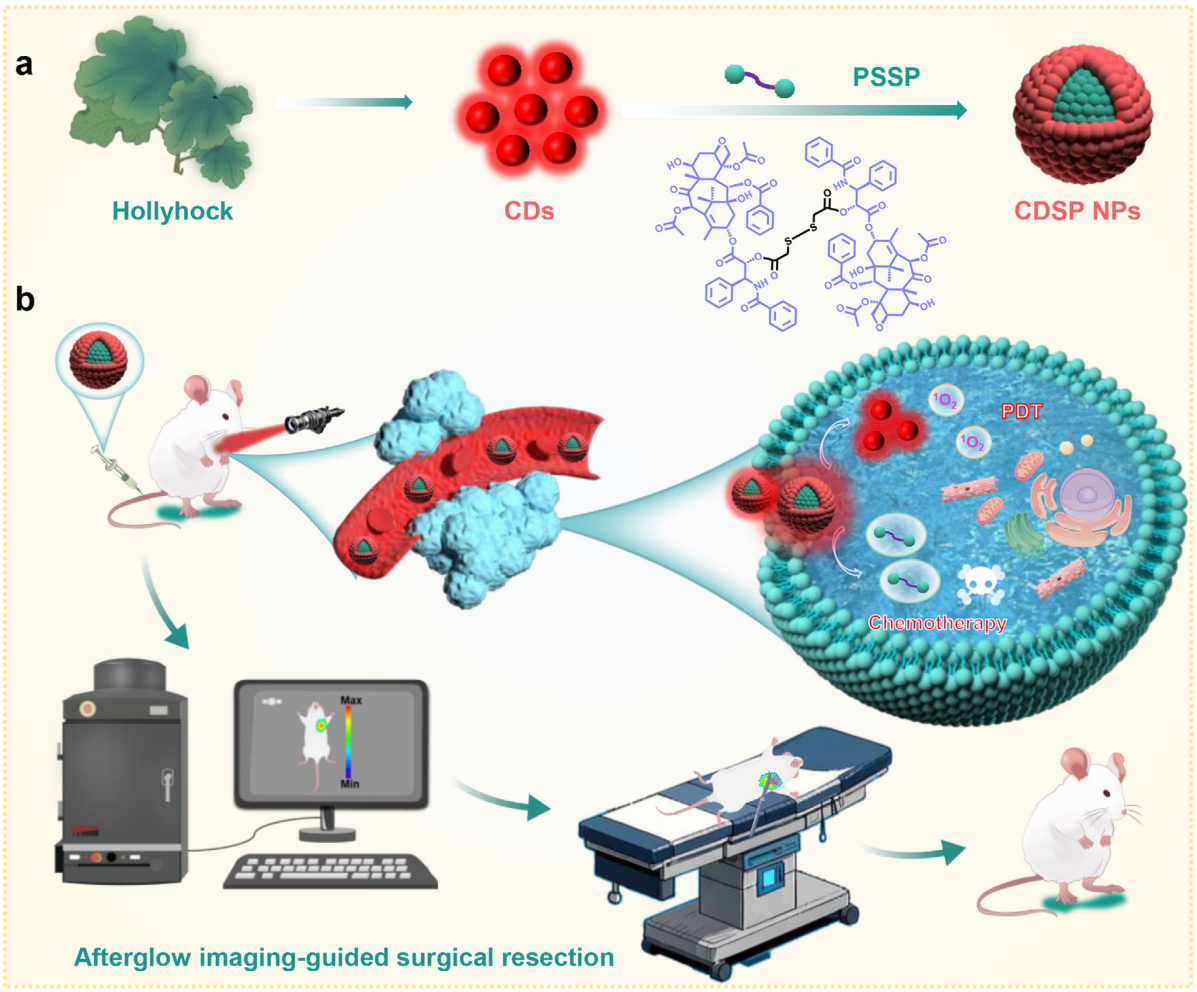
a) Preparation of CDs and CDSP NPs and b) the application of CDSP NPs for NIR afterglow imaging‐guided tumor resection and synergistic photodynamic‐chemotherapy.

Herein, CDs exhibiting NIR fluorescence and phosphorescence, along with photodynamic activity, were synthesized from hollyhock leaves through a solvothermal method. Subsequently, CDs were assembled with a disulfide‐bridged paclitaxel (PTX) prodrug (PSSP)^[^ ^24^ ^]^ to fabricate CDSP nanoparticles (NPs) (**Scheme** **1**a). The CDSP NPs are capable of furnishing real‐time intraoperative NIR afterglow imaging guidance for the complete surgical resection of tumor tissues (Scheme 1b), possessing a long lifetime (> 2 h) and a high signal‐to‐noise ratio (SNR, 103.9). Moreover, the CDSP NPs can concurrently deliver CDs and PTX to the tumor sites for synergistic photodynamic‐chemo therapy. This innovative theranostic nanoplatform presents a promising strategy for precise tumor resection and treatment.

## 2 Results and Discussion

### 2.1 Preparation and Characterization of CDs

**Figure 1:**
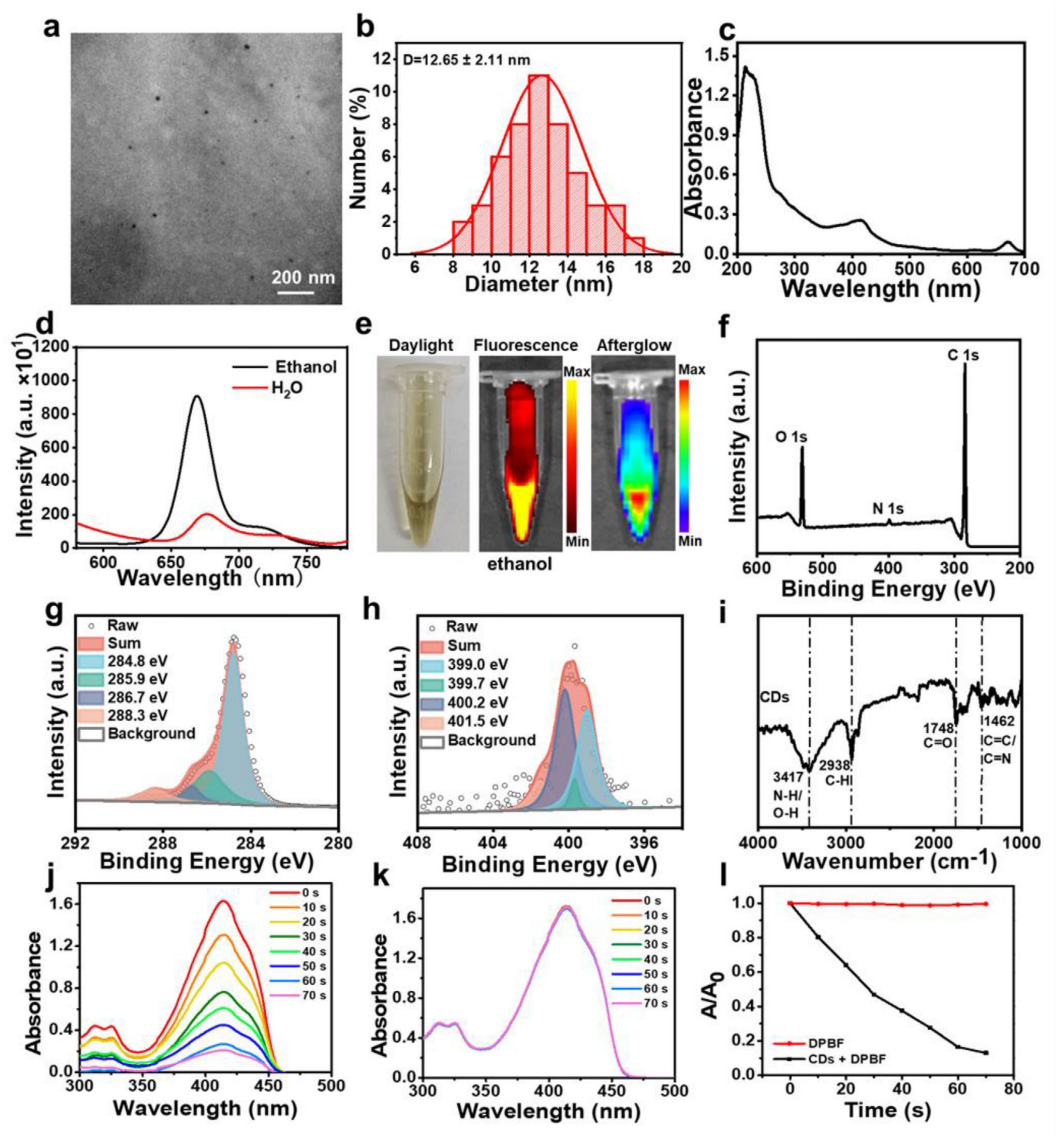
a) TEM image of CDs aqueous solution. b) Size distribution profile of CDs. c) Absorption spectrum within the UV–vis region for aqueous solutions of CDs. (10 µg mL^−1^). d) The fluorescence spectra of CDs within aqueous solution or ethanol medium. e) Visual representations including bright‐field, fluorescence and afterglow images of CDs (1 mg mL^−1^) dissolved in ethanol. f) Full survey XPS spectrum of CDs. HR g) C 1s and h) N 1s XPS spectra of CDs. i) FT‐IR spectrum of CDs. Absorption spectra of j) DPBF + CDs or k) DPBF after irradiation from 0 to 70 s using a 685 nm laser (0.1 W cm^−2^). l) Absorption variations of DPBF + CDs and DPBF at 418 nm with different irradiation time.

The solvothermal method was employed to synthesize CDs from hollyhock leaves. The Transmission Electron Microscopy (TEM) image (**Figure** **1**a) reveals that CDs have a spherical shape, and their diameter is 12.62 ± 6.11 nm (Figure 1b). The X‐ray powder diffraction (XRD) pattern of CDs (Figure S1, Supporting Information) displays two peaks. The main peak at 2θ = 20.2° corresponds to a calculated d‐spacing of approximately 0.438 nm, which is significantly larger than the interlayer spacing of the (002) plane in graphite (0.34 nm). This indicates the presence of an amorphous structure or highly defective graphitic crystallites within CDs. The secondary peak at 2θ = 40.7° corresponds to a calculated d‐spacing of approximately 0.22 nm, approaching the characteristic spacing of graphite (100) plane (ca. 0.21 nm), suggesting the presence of minor graphitic crystallites or in‐plane ordered structures. CDs display a broad absorption spectrum (Figure 1c), spanning from UV to deep‐red wavelength region, and there are three characteristic peaks centered at 214, 415, and 672 nm. The absorption peak at 214 nm can be assigned to the π‐π* transition within aromatic C = C bonds. Regarding the absorption peaks at 415 and 672 nm, they are respectively due to the n‐π* transitions of C = O and C = N bonds. The optimal excitation wavelength for CDs is 412 nm (Figure S2, Supporting Information). Under 412 nm excitation (Figure 1d), the maximum emissions of CDs in ethanol and aqueous solutions are 670 and 676 nm, respectively. Nonetheless, the fluorescence intensity of CDs in ethanol is significantly higher compared to that in an aqueous solution. The fluorescence and afterglow images of CDs were obtained. The ethanol solution of CDs is capable of emitting intense fluorescence and afterglow (Figure 1e). In contrast, the aqueous solution of CDs merely exhibits weak fluorescence, and no afterglow is observed (Figure S3, Supporting Information).

The X‐ray photoelectron spectroscopy (XPS) spectrum of CDs was collected to analyze their elemental composition and chemical bonding states (Figure 1f). Prominent peaks for C (284.8 eV), N (399.5 eV), and O (532.5 eV) were detected, confirming the presence of these elements. The HR C 1s spectrum (Figure 1g) uncovered multiple carbon species. These included C‐C/C = C at 284.8 eV, C‐N at 285.9 eV, C‐O at 286.7 eV, and C = O at 288.3 eV. In the HR N 1s spectrum (Figure 1h), peaks corresponding to pyridine‐N at 399.04 eV, N‐H band at 399.7 eV, pyrrole‐N at 400.2 eV, and graphite‐N at 401.5 eV were recognized. Regarding the HR O 1s spectrum (Figure S4, Supporting Information) peaks for C = O at 530.9 eV and C‐OH at 532.3 eV were observed, which indicated the existence of hydroxyl groups on the surface of CDs. The Fourier transform infrared spectroscopy (FT‐IR) spectrum of CDs (Figure 1i) was examined to identify characteristic absorption bands related to various functional groups. A wide‐ranging band with a center at 3417 cm^−1^ was attributed to the stretching oscillations of N‐H/O‐H bonds. The absorption occurring at 2938 cm^−1^ was associated with C‐H stretching vibrations, signifying the presence of alkyl or aromatic hydrocarbon fragments. The peak emerging at 1748 cm^−1^ was attributed to the C = O stretching vibration. The peak at 1228 cm^−1^ corresponded to the C‐N stretching vibration. Additionally, the peak at 1462 cm^−1^ was linked to the stretching vibrations of C = C/C = N bonds. These spectral characteristics offer precious perspectives on the chemical composition and bonding characteristics of the CDs.

To evaluate the ability of CDs to produce ROS, 1,3 – Diphenylisobenzofuran (DPBF) was carefully utilized as a highly sensitive indicator. When CDs were present, a notable reduction in the absorbance of DPBF at 418 nm was precisely documented (Figure 1j,l) because of the oxidative degradation of DPBF. Conversely, when DPBF was irradiated alone under the same experimental condition, its absorbance at 418 nm remained essentially unaltered throughout the irradiation period (Figure 1k,l). Based on the obtained results, a robust conclusion can be drawn that CDs exhibit a noteworthy and well‐defined ability to produce ROS upon exposure to the 685 nm laser irradiation. Furthermore, these results conclusively demonstrate that CDs exhibit both superior luminescent afterglow properties and a significant potential for the generation of ROS. These two features render CDs extremely promising contenders for numerous biomedical applications. The afterglow emission can be utilized for imaging and tracking purposes, while ROS generation ability could potentially be harnessed for cancer therapy. Electron spin resonance (ESR) spectroscopy employing 2,2,6,6‐tetramethylpiperidine (TEMP) as a spin‐trapping agent confirmed the generation of singlet oxygen (^1^O_2_) by CDs under 685 nm laser irradiation (0.1 W cm^−2^). As depicted in Figure S5 (Supporting Information), a characteristic triplet ESR signal was observed exclusively in the CDs + L group, while no significant signals were detected in the CDs + L + Vitamin C or CDs control groups. These results demonstrate the effective scavenging of ^1^O_2_ by vitamin C and exclude endogenous ROS production in the absence of light. The findings align with complementary ^1^O_2_‐specific detection using DPBF as a chemical probe. The combined use of direct ESR analysis and chemical probe validation confirms that CDs generate ^1^O_2_ via a PDT mechanism under 685 nm irradiation.

### 2.2 Preparation and Characterization of CDSP NPs

**Figure 2:**
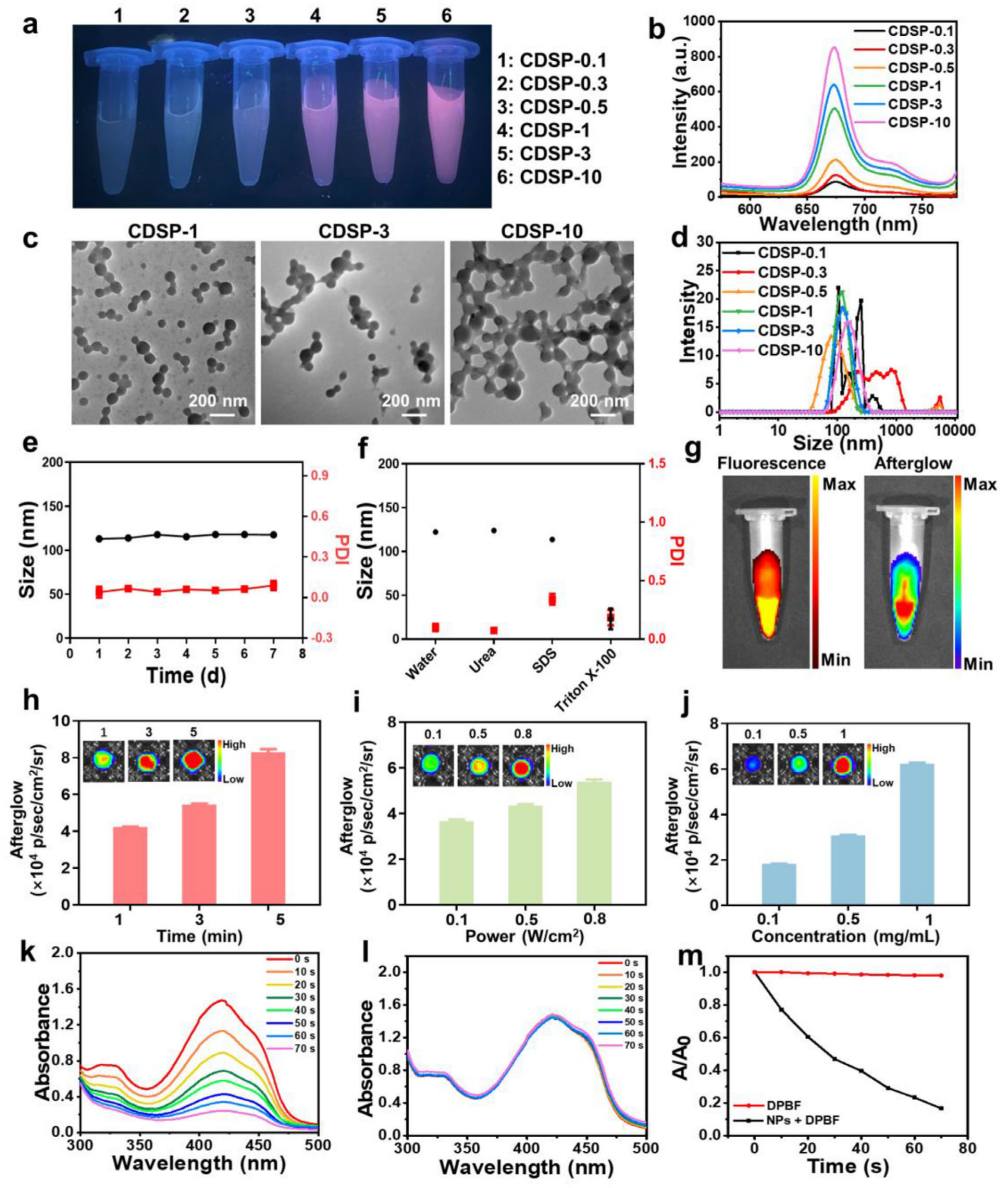
a) The photos of CDSP‐0.1, CDSP‐0.3, CDSP‐0.5, CDSP‐1 (CDSP), CDSP‐3 and CDSP‐10 NPs aqueous solution under 365 nm UV lamp (CDs: 100 µg mL^−1^). b) The fluorescence spectra of CDSP‐0.1, CDSP‐0.3, CDSP‐0.5, CDSP, CDSP‐3 and CDSP‐10 NPs aqueous solution. c) TEM images of CDSP, CDSP‐3 and CDSP‐10 NPs. d) Size distribution of CDSP‐0.1, CDSP‐0.3, CDSP‐0.5, CDSP, CDSP‐3 and CDSP‐10 NPs. e) Alterations in the size of CDSP NPs in deionized water over the course of a week. f) Variations in both size and polydispersity index (PDI) of CDSP NPs upon exposure to deionized water, urea, SDS, and Triton X‐100 solution. (*n* = 3). g) Fluorescence (at λex = 400 nm, λem = 680 nm) and afterglow (following irradiation with a 685 nm laser at 0.8 W cm⁻^2^ for 1 min) images of CDSP NPs (CDs: 1 mg mL^−1^). h) Afterglow snapshots of CDSP NPs (CDs: 1 mg mL^−1^) irradiated by a laser (685 nm, 0.8 W cm⁻^2^) at diverse time points. i) Afterglow pictures of CDSP NPs under laser illumination with varying power densities for 3 min. j) Afterglow visuals of CDSP NPs (at concentrations of 0.1, 0.5 and 1.0 mg mL^−1^) irradiated by a laser (0.8 W cm⁻^2^) for 3 min. Absorption spectra of k) DPBF + CDSP NPs or l) DPBF after irradiation from 0 to 70 s using a 685 nm laser (0.1 W cm⁻^2^). m) Absorption variations of DPBF + CDSP NPs and DPBF at 418 nm with different irradiation time.

The disulfide‐bridged paclitaxel (PTX) prodrug (PSSP) was synthesized through the conjugation of two PTX molecules with 2, 2′‐dithiodiacetic acid. Subsequently, the nanotheranostic platform (CDSP NPs) was fabricated by assembling CDs with PSSP. To optimize the reaction conditions, mass ratios of CDs to PSSP, specifically 1:0.1, 1:0.3, 1:0.5, 1:1, 1:3, and 1:10, were employed to prepare CDSP‐0.1, CDSP‐0.3, CDSP‐0.5, CDSP‐1 (CDSP), CDSP‐3, and CDSP‐10 NPs, respectively, via the nanoprecipitation method (see Supporting Information). As illustrated in **Figure** **2**a,b; Figure S6 (Supporting Information), with an increasing proportion of PSSP, the red fluorescence emitted by CDSP, CDSP‐3, and CDSP‐10 NPs was progressively enhanced. Subsequently, the morphologies of CDSP, CDSP‐3, and CDSP‐10 NPs were examined using TEM. As demonstrated in Figure 2c, CDSP NPs exhibited the most favorable dispersion, with an average diameter of 100.7 ± 15.8 nm. The hydrodynamic diameter of CDSP NPs gauged via dynamic light scattering (DLS) measurement was 114.8 ± 1.1 nm (Figure 2d). We further investigated the stability of CDSP NPs in deionized water, 10% FBS‐supplemented PBS (pH 7.4), RPMI 1640, or 5% glucose solution. The average size of CDSP NPs remained nearly unchanged (Figure 2e; Figure S7, Supporting Information), thereby validating their robust stability in physiological media. To elucidate the assembly mechanism of CDs and PSSP, CDSP NPs were treated with deionized water, urea, sodium dodecyl sulfate (SDS), and Triton X‐100 solution, respectively, and the size change was monitored by DLS. As depicted in Figure 2f, CDSP NPs dissociated after incubation with Triton X‐100, confirming that hydrophobic interaction predominated in the assembly process.

Under bioluminescent conditions, the afterglow spectra of CDs and CDSP NPs both exhibited distinct peaks at 670 nm (Figure S8, Supporting Information), highlighting the robustness of the CDs‐based nanoplatform in near‐infrared afterglow imaging.^[^ ^25^ ^]^ Fluorescence and afterglow images of CDSP NPs were obtained by using the PerkinElmer IVIS in vivo imaging system. CDSP NPs exhibited bright fluorescence and afterglow (Figure 2g). Subsequently, the effects of laser (685 nm) irradiation time and laser power on the phosphorescence of CDSP NPs were investigated. Figure 2h showed the afterglow intensity was augmented with increasing laser irradiation time. The afterglow became more intense as the power density was raised from 0.1 to 0.8 W cm^−2^ (Figure 2i). Additionally, the afterglow intensity was found to increase in direct proportion to the concentration of CDSP NPs (Figure 2j). These results indicated that the afterglow intensity was directly proportional to the irradiation time, power density, and the concentration of CDSP NPs.

DPBF served as a probe for evaluating the ROS‐generation ability of CDSP NPs. Upon irradiation with a 685 nm laser for 70 s, the absorbance at 418 nm in the DPBF + CDSP NPs group was markedly diminished (Figure 2k). In contrast, the absorbance in the DPBF group remained essentially unchanged (Figure 2l). The absorption of DPBF + CDSP NPs was significantly lower compared to that of DPBF alone (Figure 2m), suggesting that CDSP NPs possess remarkable photodynamic activity. The excellent fluorescence and phosphorescence characteristics of CDSP NPs, coupled with their remarkable ability to generate ROS, position them as highly promising candidates for applications in real‐time intraoperative imaging, precise surgical resection, and cancer therapy.

### 2.3 In Vitro Optical Imaging and Cytotoxicity of CDSP NPs

**Figure 3:**
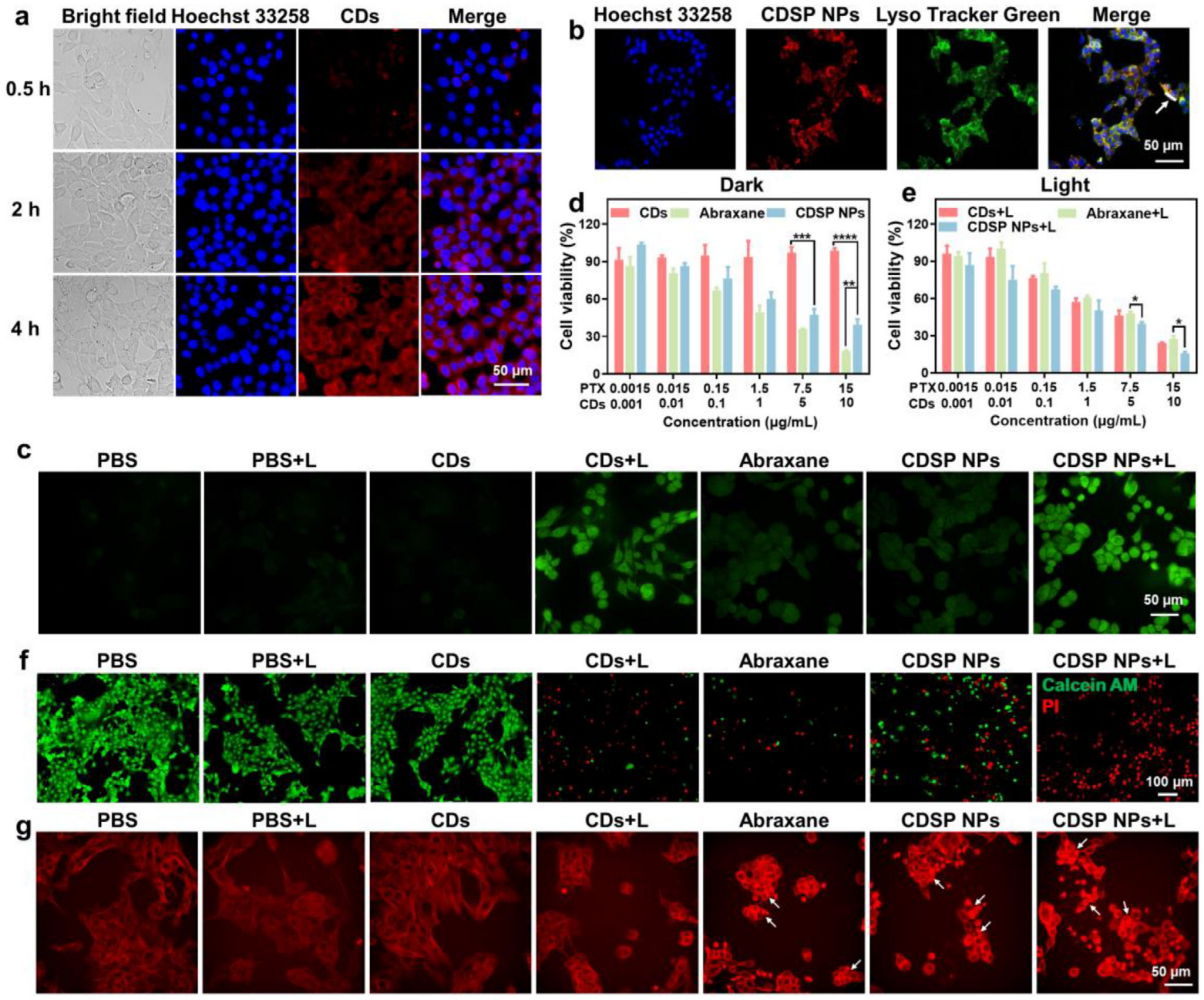
a) The fluorescence images of 4T1 cells after incubation with CDSP NPs for 0.5, 2, and 4 h. b) The fluorescence images of 4T1 cells after treated with CDSP NPs and subsequent stained with Lyso‐Tracker Green. c) The fluorescence images reflecting photo‐induced intracellular ROS generation in 4T1 cells (with CDs/PTX at a concentration of 10/15 µg mL^−1^, irradiated by 685 nm light at 0.4 W cm^−2^ for 5 min). d) The in vitro cytotoxicity of CDs, Abraxane and CDSP NPs against 4T1 cells without laser irradiation, and e) with laser irradiation (685 nm, 0.4 W cm^−2^, 5 min). f) Fluorescence images of 4T1 cells co‐stained with calcein‐AM/PI after diverse treatments (CDs/PTX: 10/15 µg mL^−1^, 685 nm, 0.4 W cm^−2^, 5 min). g) Microtubule staining images of 4T1 cells after being treated with CDs, CDs + L, Abraxane, CDSP NPs and CDSP NPs + L (CDs/PTX: 5/7.5 µg mL^−1^, 685 nm, 0.4 W cm^−2^, 5 min) were examined. Bars express SD (*n* = 3), statistical P‐values: *P < 0.1, **P < 0.01, ***P < 0.001, ****P < 0.0001.

We evaluated the cellular uptake of CDSP NPs via the incubation with 4T1 cells and subsequently the cells were observed (**Figure** **3**a). The red fluorescence within the cytoplasm progressively intensified as the incubation time was extended, indicating a temporal dependence in the endocytosis process of CDSP NPs. Then, we conducted the co‐localization experiment to assess the specificity of CDSP NPs toward lysosomes in 4T1 cells. As expected, the red fluorescence emission of CDSP NPs exhibited a substantial degree of colocalization with LysoTracker Green (Figure 3b; Figure S9, Supporting Information), and the corresponding Pearson's co‐localization coefficient was as high as 0.96. The results demonstrated that CDSP NPs mainly localized into lysosomes during the process of endocytosis in 4T1 cells.

The ROS generating capacities of CDs and CDSP NPs were assessed by using DCFH‐DA as a probe (Figure 3c). No significant fluorescence was detected in the PBS, PBS + L, CDs, Abraxane, and CDSP NPs groups. In contrast, strong green fluorescence was observed in the CDs + L and CDSP + L groups, suggesting that CDs as well as CDSP NPs possessed high photodynamic activity and were capable of generating ROS in cells. We further performed quantitative detection of ROS levels in 4T1 cells using flow cytometry combined with the DCFH‐DA probe (Figure S10, Supporting Information). The results showed that the positive level of the CDSP NPs + L treatment group was 4.3‐fold that of the PBS treatment group. Compared with the CDs + L group, the CDSP NPs + L group exhibited higher fluorescence intensity, a phenomenon indicating that CDSP NPs significantly enhanced intracellular oxidative stress through the synergistic effect of photodynamic therapy and chemotherapy. Subsequently, to explore the in vitro cytotoxicity of CDs, Abraxane, and CDSP NPs towards 4T1 cells, the 3‐(4,5‐dimethylthiazol‐2‐yl)‐2,5‐diphenyltetrazolium bromide (MTT) assay was utilized. In the absence of laser exposure, the cell viability in the CDs group was nearly 100%, indicating the favorable cytocompatibility of CDs (Figure 3d). By comparison, the survival rate of 4T1 cells was 39.6 ± 4.3% after treatment with CDSP NPs at an equivalent dose of CDs (10 µg mL^−1^), demonstrating that PSSP in CDSP NPs led to the death of approximately 60% of 4T1 cells. In the presence of laser irradiation (Figure 3e), the cell viability in the CDs + L group was 24.4 ± 0.2%, validating the effective photodynamic activity of CDs. The survival rate of 4T1 cells in the CDSP + L group declined to 18.8 ± 1.3%, confirming that CDSP NPs achieved a synergistic therapeutic effect through photodynamic‐chemo combination therapy. Subsequently, a live‐dead cell staining assay was carried out to appraise the combined therapeutic effectiveness of CDSP NPs in conjunction with Calcein‐AM and propidium iodide (PI). Although both green and red fluorescence were simultaneously present in the CDs + L and CDSP NPs groups (Figure 3f), the evidently intense red fluorescence in the CDSP NPs + L group further corroborated the synergistic photodynamic‐chemo efficacy of CDSP NPs. Additionally, to verify the contribution of chemotherapy, the anti‐cancer mechanism of PTX was investigated by immunofluorescence staining of microtubule proteins (Figure 3g). It was discovered that only the PTX‐containing groups (Abraxane, CDSP NPs, and CDSP NPs + L) exhibited different numbers of microtubule bundles, confirming that PSSP in the CDSP NPs + L induced cell death.

### 2.4 In Vivo Optical Imaging

**Figure 4:**
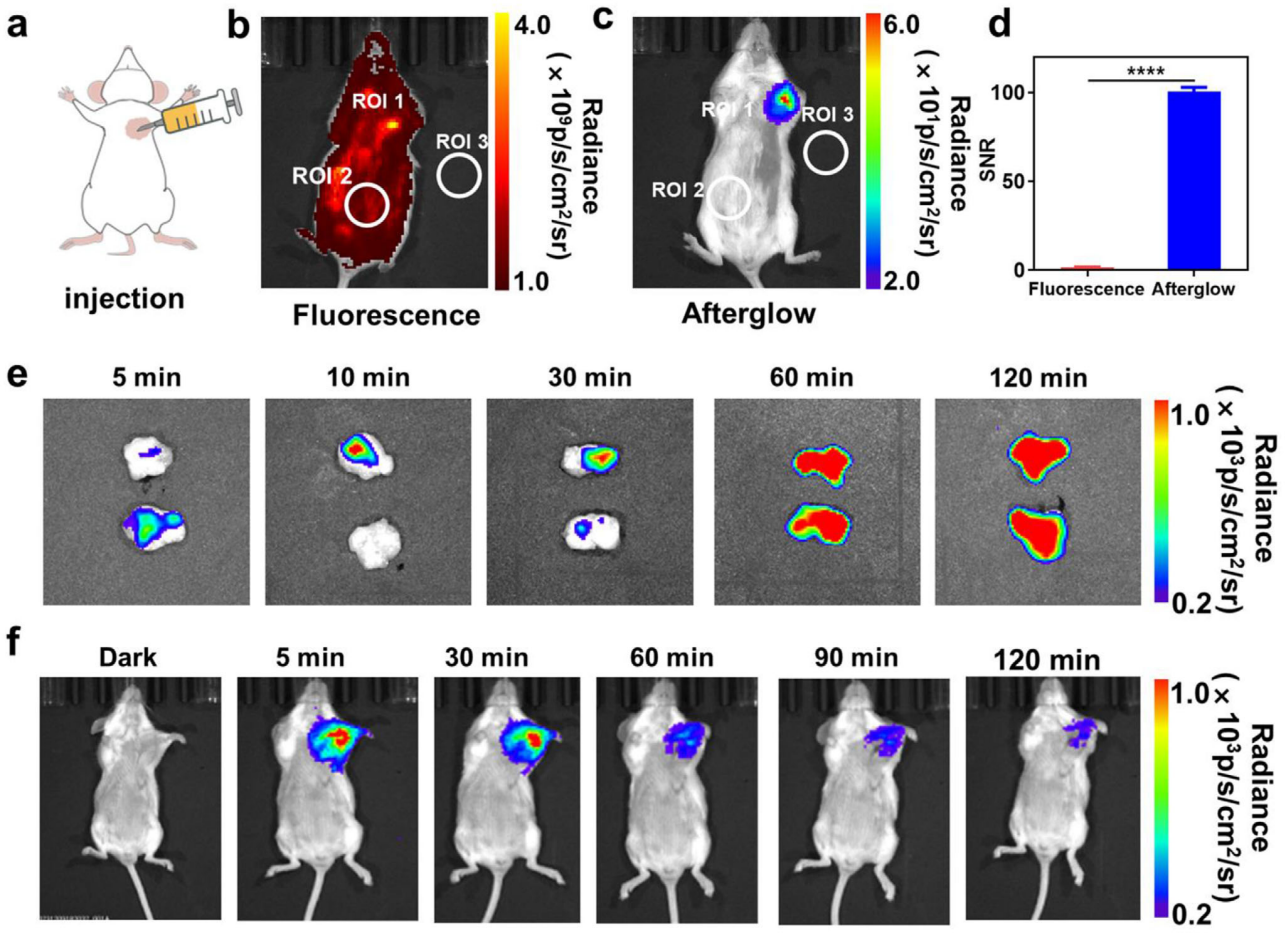
a) Diagram of afterglow imaging in live mice. b) Fluorescence and c) afterglow imaging results of mice subsequent to intratumoral injection of CDSP NPs (with a concentration of CDs/PTX: 15/22.5 mg mL^−1^, volume of 50 µL). d) Quantitative SNR values derived from the images in (b) and (c). e) Maximum cross‐sectional afterglow images of 4T1 tumors post‐injection of CDSP NPs, following illumination (685 nm, 0.8 W cm⁻^2^, 5 min). f) Afterglow snapshots of the tumor after the injection of CDSP NPs (CDs/PTX: 15/22.5 mg mL^−1^, 50 µL). Bars express SD (*n* = 3), statistical P‐values: ****P < 0.0001.

CDSP NPs were injected intratumorally into the axillary region of mice (**Figure** **4**a). By employing the IVIS in vivo imaging system under the fluorescence modality, fluorescent images were obtained. A feeble fluorescent signal was detected at the tumor location (Figure 4b). Subsequently, CDSP NPs were triggered for 5 min using a 685 nm laser at a power intensity of 0.8 W cm^−2^. After that, luminescent images were captured in the bioluminescence mode, and a strong, persistent luminescence signal was detected at the tumor site (Figure 4c,d). The quantitative SNR of the luminescence was calculated to be 103.9, which was 61.1 times greater than that of the fluorescence signal. This finding thereby validated the promise of CDSP NPs for in vivo afterglow imaging.

To explore the accumulation of CDSP NPs at the tumor tissues, we conducted luminescence imaging at multiple time points after intratumoral injection. As depicted in Figure 4e, 60 min post‐injection, the luminescent signals of the entire cross‐section of the tumors were observed, indicating that CDSP NPs could accumulate and disperse throughout the tumor tissues in 60 min. Next, CDSP NPs were introduced into 4T1 tumor‐bearing mice. Thereafter, the aforesaid mice were subjected to irradiation using a 685 nm laser at a power intensity of 0.8 W cm^−2^ for a duration of 10 min. A pronounced and persistent luminescence signal was discerned at the tumor location, and it continued to be observable even 120 min post‐irradiation (Figure 4f), suggesting that CDSP NPs possessed a long afterglow lifetime. Furthermore, we delved deeper into the sustainability of CDSP NPs. Although the luminescence signal disappeared after 120 min, it could be recharged after 10 min of laser irradiation (Figure S11, Supporting Information). After three cycles of recharging, the intensity of luminescence remained nearly unchanged. Such beneficial rechargeable persistent luminescence is favorable for attaining prolonged in vivo imaging. The afterglow tissue penetration depth of CDSP NPs was explored by positioning a mouse above a pre‐irradiated CDSP NPs solution (Figure S12, Supporting Information). Even with a 13‐mm overlay of mouse tissue, the luminescent signal could still be detected, indicating the penetrating advantages of afterglow imaging.

### 2.5 Optical Imaging‐Guided Resection of 4T1 Tumors

**Figure 5:**
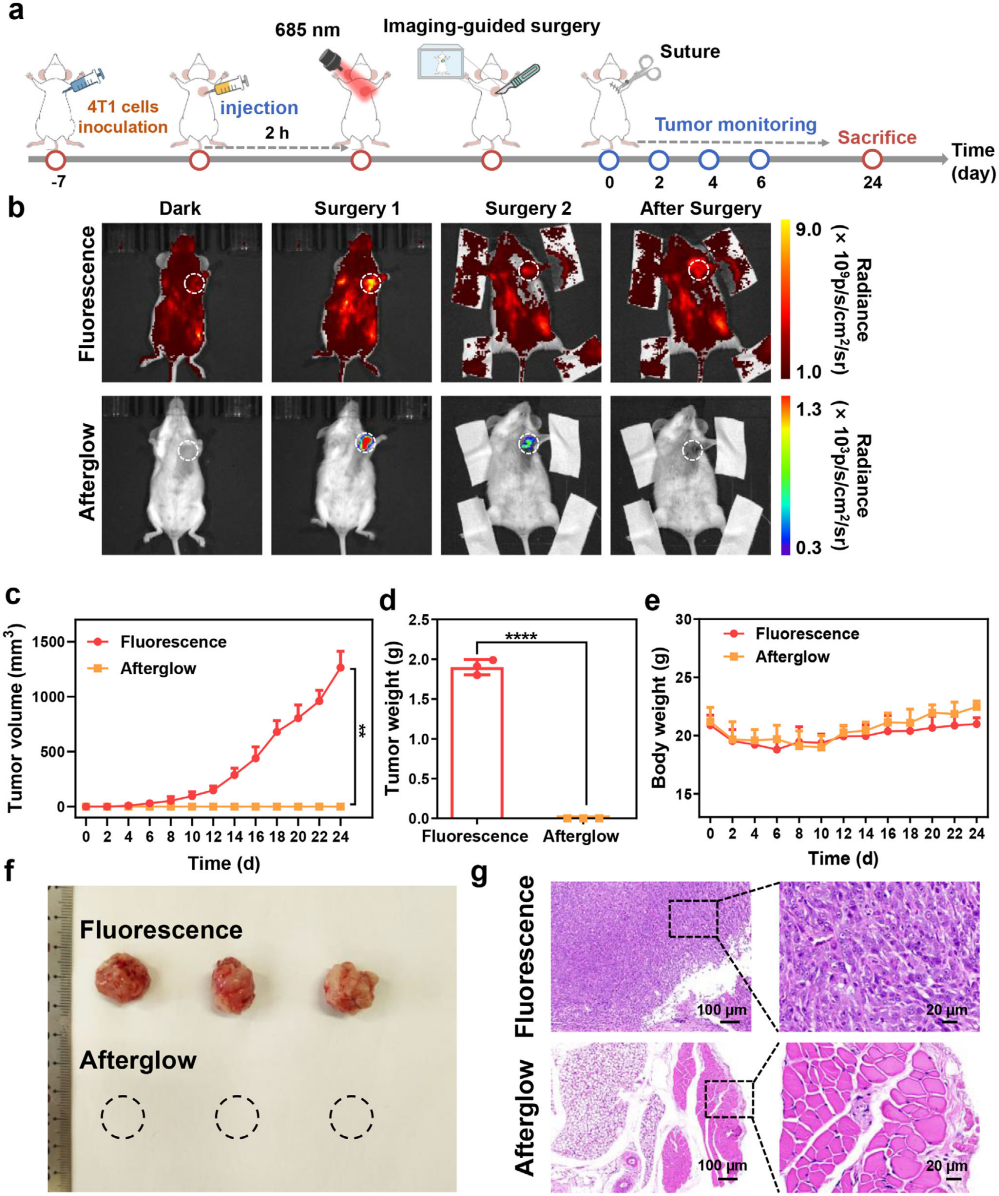
a) The schematic illustration of optical imaging guided surgical resection of tumors. b) Fluorescence and afterglow imaging during the surgical resection process (white circles indicate the tumor sites). c) The tumor volume, d) tumor weight and e) body weight of mice in the fluorescence and afterglow imaging groups. f) The photos of tissues collected from the resection sites on the day 24. g) H&E staining of the tumor margin on the day 24. Data express as Mean ± SD (*n* = 3). **P < 0.01, ****P < 0.0001.

In light of the high‐contrast afterglow imaging in tumor tissues, we simulated the clinical surgical process and carried out a near‐infrared (NIR) optical imaging‐assisted tumor resection. A subcutaneous 4T1‐tumor xenograft model was established in Balb/c mice (**Figure** **5**a). Upon the tumor size attaining around 100 mm^3^, the mice were randomly divided into two groups, designated for fluorescence imaging and afterglow imaging correspondingly. CDSP NPs were administered intratumorally to the Balb/c mice, and surgical resection was carried out 2 h following the injection. Preoperative fluorescence or afterglow imaging was conducted to determine the tumor location, thereby guiding the first surgical resection. After the first resection, fluorescence imaging was repeated to identify any tumor remnants for the second precise resection. Upon completion of the second surgical resection, imaging was performed on the mice to ensure that no optical signals were detected, signifying that all of the tumor tissues had been removed. As depicted in Figure 5b, the surgical resection guided by fluorescence imaging was severely affected by strong autofluorescence interference. In contrast, the cancerous region exhibited a high NIR luminescent SNR and could be easily dissected under the guidance of afterglow imaging. With the assistance of the phosphorescence signal, the tumor margin was accurately defined, ensuring precise excision during the subsequent surgery. After two resections, no afterglow signal was observed, indicating that the tumor tissue had been completely eradicated.

Following two surgical resections, the wounds were sutured and monitored regularly for 24 days. In the fluorescence imaging group, tumor recurrence was observed, with the tumor volume (Figure 5c) and tumor weight (Figure 5d) gradually increasing. By contrast, the tumor tissue within the afterglow imaging group was thoroughly eliminated, and as a result, there was no reappearance of the tumor. Simultaneously, the body weight of the mice from both groups was monitored and documented, and no significant abnormality was detected (Figure 5e), indicating that the NIR imaging‐guided surgical resections had no obvious adverse effects on the mice. Furthermore, on day 24, the tissues from the surgical resection sites were collected (Figure 5f) and subjected to H&E staining (Figure 5g). In the incisional margin tissue of the mice belonging to the fluorescence imaging group, a substantial quantity of tumor cells could be discerned. In contrast, within the tissue section of the afterglow imaging group, not a single tumor cell was detected, confirming the precise tumor resection guided by the afterglow imaging.

### 2.6 In Vivo Photodynamic‐Chemo Therapy and Biosafety of CDSP NPs

**Figure 6:**
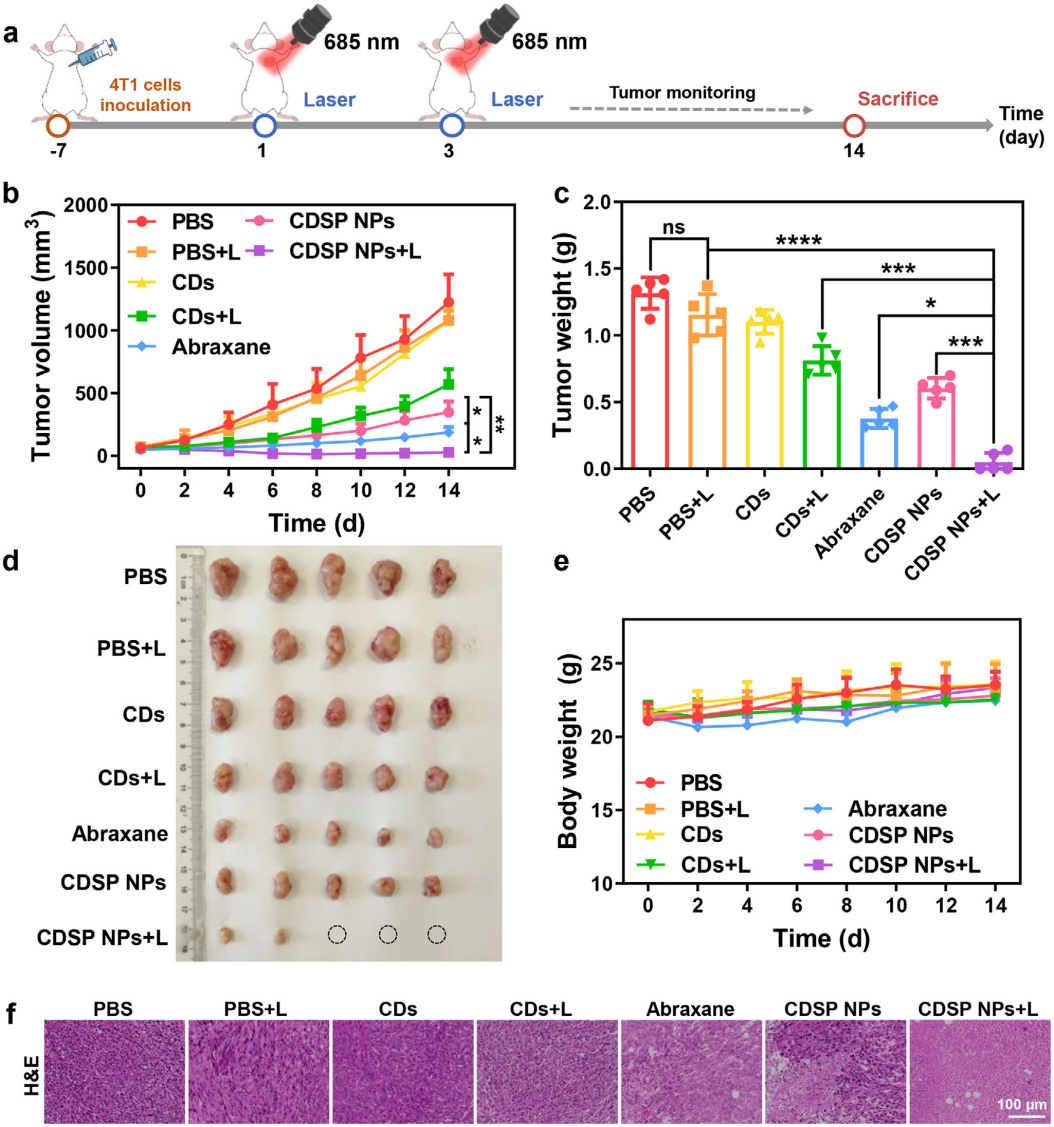
a) Illustration of animal treatment protocols. b) Tumor volume, c) Tumor weight, d) Tumor photos, e) Body weight of mice and f) H&E staining of tumors in the PBS, PBS + L, CDs, CDs + L, Abraxane, CDSP NPs, and CDSP NPs + L groups. Data express as Mean ± SD (*n* = 5). *P < 0.1, **P < 0.01, ***P < 0.001, ****P < 0.0001.

Subsequently, the synergistic therapeutic efficacy was investigated. At the axillary area of Balb/c mice, a subcutaneous xenograft tumor model of 4T1 was successfully constructed (**Figure** **6**a). Upon reaching a tumor volume of around 80 mm^3^, the murine subjects were randomly sorted into seven distinct groups, namely PBS, PBS + L, CDs, CDs + L, Abraxane, CDSP NPs, and CDSP NPs + L. Subsequently, the corresponding formulations were intravenously injected at equal CDs/PTX doses of 10/15 mg/kg on days 1 and 3, and irradiated (0.3 W cm^−2^, 10 min) 12 h after injection. During the treatment period, the tumor dimensions and the body weights of the mice were gauged at two‐day intervals. The tumor size in the PBS, PBS + L, and CDs groups was significantly enlarged (Figure 6b), suggesting that either CDs or laser irradiation alone was incapable of inhibiting tumor growth. Simultaneously, post‐treatment with CDs + L, Abraxane, or CDSP NPs, tumor growth was mitigated variably, attributable to either single PDT or chemotherapy. In contrast, the CDSP NPs + L group exhibited the most potent tumor suppression. At the conclusion of the 14‐day treatment protocol, the mice underwent euthanasia, after which both tumor tissues and major organs were harvested for further analysis. Among the seven groups, the CDSP NPs + L group exhibited the lowest tumor weight (Figure 6c). Remarkably, in this group, three tumors were entirely eliminated (Figure 6d). H&E staining of tumor tissue sections further verified that the treatment with CDSP NPs + L led to the most conspicuous tumor cell apoptosis (Figure 6f). This result further substantiated the enhanced anti‐tumor efficacy of CDSP NPs because of the synergistic PDT and chemotherapy.

Furthermore, the biosafety of CDSP NPs was appraised through the surveillance of body weight fluctuations and the performance of routine hematological assays in murine models. The mice demonstrated a tendency of slow weight gain, indicating that CDSP NPs had minimal side effects (Figure 6e). Analogous to the control group, the hematological parameters within the CDSP NPs + L group remained within the normal physiological range (Figure S13, Supporting Information). H&E staining of major organs (Figure S14, Supporting Information) revealed that there were no significant histopathological aberrations or lesions in the CDSP NPs + L group. Collectively, these results demonstrated that the CDSP NPs + L treatment did not induce conspicuous systemic toxicity or hepatorenal function impairment.

## 3 Conclusion

To sum up, we have demonstrated that CDSP NPs represent an ideal nanoplatform for efficient afterglow imaging‐guided tumor resection and synergistic photodynamic‐chemo therapy. The NIR afterglow imaging of CDSP NPs exhibits the advantages of a long lifetime, deep tissue penetration, a high SNR, and precise localization within tumor sites. These characteristics facilitate the complete and safe surgical excision of breast cancer, and enable the effective suppression of cancer recurrence. Furthermore, CDSP NPs are capable of efficiently generating ROS for photodynamic therapy and releasing PTX for chemotherapy. In vitro and in vivo investigations have verified that CDSP NPs can be utilized for the inhibition or even elimination of tumors through synergistic photodynamic‐chemo treatment, with relatively fewer adverse effects. It is widely acknowledged that this innovative approach has proffered an incisive concept and valuable application, which are of great significance for remarkably augmenting imaging‐guided tumor resection and treatment.

## 4 Experimental Section

### Synthesis of CDs

The fresh leaves of hollyhock were collected and dried at 65 °C to eliminate water. Subsequently, 1.0 g of dried leaves and 10 mL of ethanol solution were transferred into poly(tetrafluoroethylene)‐lined autoclaves and heated at 160 °C for 4 h. After cooling to room temperature, the supernatant solution was filtered through a 0.45 µm polyethersulfone membrane to remove large particles. Finally, the solution was dried to obtain CDs.

### Preparation of CDSP NPs

CDSP NPs were fabricated using the nanoprecipitation method. One milligram of CDs and 1 mg of PSSP were dissolved in an ethanol solution (1 mL), and then the solution was added dropwise into deionized water (10 mL) with vigorous stirring for overnight. CDSP NPs were purified by dialysis and centrifugation. The mass ratio of CDs to PSSP in the obtained CDSP NPs was determined to be 1:1.5 by UV–vis absorption spectra. Additionally, CDSP NPs‐0.1, CDSP NPs‐0.3, CDSP NPs‐0.5, CDSP NPs‐3, and CDSP NPs‐10 NPs were prepared in a manner similar to the preparation of CDSP NPs.

### Afterglow Imaging‐Guided Resection of 4T1 Tumor

All animal experiments were approved by the Animal Welfare and Ethics Committee of Changchun Institute of Applied Chemistry, Chinese Academy of Sciences (Approval No. 2023‐0132). Balb/c mice with a tumor size of approximately 100 mm^3^ were randomly divided into two groups. CDSP NPs (at a CDs/PTX ratio of 15/22.5 mg mL^−1^, 50 µL) were injected intratumorally into the Balb/c mice, and surgical resection was carried out 2 h later under the guidance of fluorescence or afterglow imaging. Preoperative fluorescence or afterglow imaging was performed to determine the tumor location and guide the first surgical resection. After the first resection, fluorescence or luminescence imaging was repeated to identify any tumor remnants for a second precise excision. Upon completion of the second resection, imaging was conducted on the mice to ensure that no luminescent signal was detected, signifying that all tumor tissue had been removed. At this stage, the wound was sutured and monitored regularly. The body weight and tumor volume of the mice were measured every two days. After 24 d following the surgical resection, the mice were sacrificed and the tumors were collected to evaluate the tumor recurrence rate.

### Statistical Analysis

Data were expressed as mean ± standard deviation (SD). One‐way or two‐way analysis of variance (ANOVA) was used to determine the statistical significance. Data with p < 0.05 were considered significant, and the analysis was performed using GraphPad Prism 8 software.

## Conflict of Interest

The authors declare no conflict of interest.

## Supporting information



Supporting Information

## Data Availability

The data that support the findings of this study are available in the supplementary material of this article.
