# Supplementary material for: Near‐Infrared Afterglow Imaging‐Guided Surgical Resection and Synergistic Photodynamic‐Chemo Therapy of Breast Cancer
Source: Adv Sci (Weinh). 2025 Jun 10;12(33):e03883. doi: 10.1002/advs.202503883 (PMC12412500; doi:10.1002/advs.202503883)
Supplement: Supplementary file 1 — Supporting Information [file ADVS-12-e03883-s001.docx]

Supporting Information

Near-Infrared Afterglow Imaging-Guided Surgical Resection and Synergistic Photodynamic-Chemo Therapy of Breast Cancer

Zixuan Li, ^#^ Ranran Zhao, ^#^ Qing Pei, Zhigang Xie, * and Min Zheng *

**Materials and characterization:**

3-[4,5-Dimethylthiazol-2-yl]-2,5-diphenyltetrazolium bromide (MTT) was procured from Genye Biotechnology Co. Ltd (Shanghai, China). Cell culture dishes and centrifuge tubes were obtained from NEST Biotechnology Co. Six-well plates and 96-well plates were purchased from Guangzhou JITE Biofiltration Co. Lysosomal localization probe Lyso-Tracker Green, Calcein/PI, cell viability/cytotoxicity assay kit, and microtubule protein-Tracker Red were acquired from Shanghai Biyuntian Biotechnology Co.

Transmission electron microscopy (TEM) images were captured by a JEOL JEM-1011 (Japan) at an accelerating voltage of 100 kV. UV-visible absorption spectra were measured on a Shimadzu UV-2450 spectrophotometer. Fourier transform infrared (FT-IR) spectra of CDs were recorded within the range of 4000 to 500 cm^-1^ on a TJ270-30A infrared spectrophotometer of Tianjin Jing tuo Instrument Science and Technology Co. The Zetasizer Nano-ZS (Malvern Instruments Ltd.) was employed to analyze the size of CDSP NPs. Endocytosis, lysosomal localization, and microtubule images were obtained using an Operetta CLS™ high content analysis system (PerkinElmer, USA). *In vivo* and *in vitro* optical imaging was carried out using the PerkinElmer IVIS Lumina Seres III *in vivo* imaging system (PerkinElmer, USA).

**Synthesis of CDs**

Fresh hollyhock leaves were rinsed with deionized water to remove surface impurities, air-dried, and placed into a pre-cooled mortar. Liquid nitrogen was added, and the leaves were rapidly ground into a uniform powder. The ground leaf powder was transferred to an oven and dried at 65 ^o^C for 30 min to completely remove moisture. Subsequently, 1.0 g of the dried leaf powder and 10 mL of ethanol were added to a Teflon-lined autoclave, which was sealed and heated at 160 ^o^C for 4 h. After cooling to room temperature, the reaction mixture was centrifuged to collect the supernatant (3500 rpm, 5 min), which was filtered through a 0.45 μm polyethersulfone membrane to remove large particulate impurities. The filtrate was dried to obtain solid CDs.

**Near-infrared fluorescence and afterglow imaging of CDs in water and ethanol**

The fluorescence and afterglow imaging procedures were carried out with the utilization of an IVIS imaging system, wherein the fluorescence imaging was performed in the fluorescence mode and the afterglow imaging was executed in the bioluminescence mode. For the acquisition of fluorescence images, an excitation wavelength of 400 nm was applied, and the emission wavelength was set at 680 nm. In the case of phosphorescence images, they were collected subsequent to irradiation by a 685 nm laser at a power density of 0.8 W cm^-2^ for a duration of 1 min. Subsequently, the intensities of both the fluorescence and afterglow were analyzed by means of the Living Imaging 4.3 software.

**Synthesis of PSSP**

PSSP was synthesized by bridging PTX with 2, 2'-dithiodiacetic acid according to our previous work ^[1]^.

**Preparation of CDSP NPs**

CDSP NPs were fabricated using the nanoprecipitation method. One milligram of CDs and 1 mg of PSSP were dissolved in an ethanol solution (1 mL), and then the solution was added dropwise into deionized water (10 mL) with vigorous stirring for overnight. CDSP NPs were purified by dialysis and centrifugation. The ratio of CDs to PSSP in the obtained CDSP NPs was determined to be 1:1.5 by UV-vis absorption spectra. Additionally, CDSP-0.1, CDSP-0.3, CDSP-0.5, CDSP-3, and CDSP-10 NPs were prepared in a manner similar to the preparation of CDSP NPs.

***In vitro* stability.**

To investigate the *in vitro* stability, CDSP NPs were incubated with deionized water, 5% PBS (pH 7.4) solution containing 10% fetal bovine serum (FBS), Roswell Park Memorial Institute 1640 (RPMI 1640) medium, or glucose solution, at 37 ^o^C for different durations. The changes in the hydrodynamic diameter and polydispersity index (PDI) of CDSP NPs were measured using dynamic light scattering (DLS).

**Intermolecular interactions investigation**

CDSP NPs were incubated with urea, sodium dodecyl sulfate (SDS), and Triton X-100 (0.1%), respectively, at 37 ^o^C for 3 h. The change of size and PDI was recorded by DLS.

**Reactive oxygen species (ROS) assay**

The ability of CDSP NPs to generate ROS *in vitro* was evaluated by detecting the absorption change of DPBF at 415 nm. CDSP NPs (with a CDs/PTX ratio of 10/15 μg mL^-1^) were added to the DPBF solution. The solution was then irradiated with a 685 nm laser at a power density of 0.1 W cm^-2^. The absorption spectra of the solution were recorded at 10-second intervals. A solution containing only DPBF was used as a control.

**Near-infrared fluorescence and afterglow imaging of CDSP NPs.**

Fluorescence and afterglow imaging were acquired using an IVIS imaging system in fluorescence and bioluminescence modes, respectively. Fluorescence images were obtained upon excitation at 400 nm and emission at 680 nm. Phosphorescence images were collected after irradiation with a 685 nm laser (at a power density of 0.8 W cm^-2^) for 1 min. The fluorescence and afterglow intensities were analyzed using Living Imaging 4.3 software.

**Cell culture.**

4T1 cells were cultured in 1640 medium containing 10%FBS, 1%(v/v) penicillin/streptomycin antibiotics. Cells were placed in an incubator at 37 ^o^C containing 5% CO_2_ and 95% humidified air. The cells were checked periodically for mycoplasma contamination.

**Cellular uptake.**

Cellular uptake of CDSP NPs was evaluated by the Operetta CLS^TM^ high content Analysis system (PerkinElmer, USA) in non-confocal mode. Initially, 4T1 cells were seeded into 96-well culture plates (at a density of 3 × 10^3^ cells per well) and allowed to adhere to the well surface for 24 h. Subsequently, the medium was removed, and the cells were incubated with RPMI 1640 medium supplemented with CDSP NPs (at a CDs/PTX ratio of 20/30 μg mL⁻¹) for an additional 0.5, 2, and 4 h, respectively. Samples were imaged after staining the nuclei with Hoechst 33258 for 5 min.

**Lysosomal localization**

First, 4T1 cells were seeded into 96-well plates (at a density of 3 × 10^3^ cells per well) and allowed to adhere to the well surface for 24 h. Subsequently, the medium was removed and the cells were incubated with RPMI 1640 medium supplemented with CDSP NPs (at a CDs/PTX ratio of 20/30 μg mL^-1^) at 37 ^o^C for 4 h. Thereafter, the cells were incubated with the lysosomal probe Lyso-Tracker Green for 0.5 h. Then, the nuclei of the cells were stained with Hoechst 33258 for 5 min. Subsequently, co-localization was analyzed by the Operetta CLS™ high content analysis system (PerkinElmer, USA) in the non-confocal mode.

**Cytotoxicity assay.**

4T1 cells were seeded into 96-well plates at a density of 5 × 10^3^ cells per well. One hundred microliters of RPMI 1640 medium was added, and the cells were incubated for 24 h. Subsequently, the medium was replaced with fresh medium containing different concentrations of CDs, Abraxane, and CDSP NPs. After incubation for 4 h, the cells were exposed to 685 nm laser irradiation at a power density of 0.4 W cm^-2^ for 5 min or kept in the dark. Following an additional incubation for 48 h, 20 μL of MTT was added, and the cells were further incubated at 37 ^o^C for 4 h. Then, the supernatant medium was carefully aspirated, and 150 μL of DMSO was added. Finally, the plate was shaken for 3 min, and the absorbance of the purple product at 490 nm was measured using a microplate reader.

**Intracellular ROS detection.**

Intracellular ROS production was monitored using the Operetta CLS™ high content analysis system. 4T1 cells were seeded into 96-well culture plates and cultured for 24 h. After the 24-h culture period, CDs, Abraxane, and CDSP NPs (at a CDs/PTX ratio of 10/15 μg mL^-1^, 100 μL) were added and incubated for 4 h. Subsequently, the groups designated as '+L' were irradiated with a 685 nm laser (at a power density of 0.4 W cm^-2^) for 5 min, while the dark groups were left without treatment. The supernatant was then carefully aspirated, and a DCFH-DA solution in serum-free RPMI 1640 was added and incubated with the cells for 30 min at 37 ^o^C. Fluorescence images of the DCF induced by ROS were observed using the Operetta CLS™ high content analysis system.

Intracellular ROS generation was monitored by flow cytometry. 4T1 cells were seeded into 24-well culture plates and cultured for 24 h. After 24 h of culture, CDs, Abraxane, and CDSP NPs (CDs/PTX:10/15 μg/mL, 500 μL) were added and incubated for 4 h. Subsequently, the groups labeled "+ L" were irradiated with a 685 nm laser (power density: 0.4 W cm^-2^) for 5 min. The supernatant was then aspirated, and cells were detached with trypsin and transferred to centrifuge tubes. Cells were collected by centrifugation, followed by resuspension in a DCFH-DA solution diluted with serum-free RPMI 1640. The cell suspension was incubated at 37 ^o^C for 30 min. After incubation, cells were harvested by centrifugation, the supernatant was discarded, and the cell pellet was resuspended in PBS. After another centrifugation and removal of the supernatant, cells were resuspended in 1 mL of PBS, and the fluorescence of DCF generated by ROS was quantitatively analyzed by flow cytometry.

**Calcein-AM/PI staining test.**

4T1 cells were incubated with CDs, Abraxane, and CDSP NPs (at a CDs/PTX ratio of 10/15 μg mL^-1^, 100 μL) for 4 h. Subsequently, the cells were either exposed to 685 nm irradiation for 5 min (at a power density of 0.4 W cm^-2^) or maintained in the dark. After an additional incubation for 24 h, the medium was removed, and the cells were gently washed with PBS. Subsequently, the cells were stained with a calcein-AM/PI solution in the dark for 30 min at room temperature. Finally, the samples were imaged using a fluorescence microscope.

**Microtubule staining assay.**

Microtubule protein immunofluorescence staining was employed to assess the inhibitory effect of CDSP NPs on cell proliferation. 4T1 cells were incubated with CDs, Abraxane, and CDSP NPs (at a CDs/PTX ratio of 5/7.5 μg mL^-1^, 100 μL) for 4 h. Subsequently, the cells were either exposed to 685 nm irradiation for 5 min (at a power density of 0.4 W cm^-2^) or maintained in the dark. After an additional incubation for 24 h, the medium was removed, and the cells were gently washed with PBS. Thereafter, the supernatant of the medium was removed, the cells were gently washed again, fixed with 4% paraformaldehyde (100 μL per well) for approximately 12 min, and then stained with PBS containing 0.1% Triton X-100 (pH 7.4) solution three times. The cells were then incubated with a microtubulin immunostaining secondary antibody, which was diluted with PBS (pH 7.4) solution containing 0.1% Triton X-100 and 3% BSA, for 30 min at room temperature in the dark. Subsequently, the stained cells were imaged using the Operetta CLS^TM^ high content analysis system.

**Tumor models.**

All animal experiments were approved by the Animal Welfare and Ethics Committee of Changchun Institute of Applied Chemistry, Chinese Academy of Sciences (Approval No. 2023-0132) and were conducted in accordance with the National Institutes of Health Guide for the Care and Use of Laboratory Animals (NIH Publication No. 85-23 Rev. 1985). Female Balb/c mice were procured from Liaoning Changsheng Biotechnology Co. and were maintained under specific conditions. To establish the tumor model, 4T1 cells were subcutaneously inoculated into the lateral aspect of the anterior right limb of the mice (with 8×10^5^ cells in 100 μL of PBS). The tumors were allowed to grow for 5-7 days prior to the imaging experiments.

***In vivo* tumor imaging.**

NIR fluorescent and afterglow imaging was carried out on 4T1 tumor-bearing mice using the IVIS *in vivo* imaging system. CDSP NPs (at a CDs/PTX ratio of 15/22.5 mg mL^-1^, 50 μL) were injected intratumorally into female 4T1 mice. Subsequently, the *in vivo* fluorescent images of the mice were captured under 400 nm excitation. Thereafter, CDSP NPs were irradiated with a 685 nm laser for 5 min (at a power density of 0.8 W cm^-2^), and then afterglow imaging was performed.

CDSP NPs (at a CDs/PTX ratio of 15/22.5 mg mL⁻¹, 50 μL) were injected intratumorally into the tumors. Subsequently, the tumor regions were irradiated with a 685 nm laser at a power density of 0.8 W cm^-2^ for 10 min to obtain afterglow luminescence images of the mice, which were collected at different time points.

Serial afterglow imaging was conducted by injecting CDSP NPs (at a CDs/PTX ratio of 15/22.5 mg mL^-1^, 50 μL). The tumor area was irradiated with a 685 nm laser (at a power density of 0.8 W cm^-2^) for 10 min, and the afterglow luminescence images were acquired immediately after the laser was switched off. Two hours later, the tumor area was re-irradiated with a 685 nm laser (at a power density of 0.8 W cm^-2^) for 10 min, and then the afterglow images were collected.

The tumor-bearing mice were injected intratumorally with CDSP NPs (at a CDs/PTX ratio of 15/22.5 mg mL^-1^, 50 μL), and the mice were dissected at 5, 10, 30, 60, and 120 min post-injection. After the excised tumors were irradiated with a 685 nm laser (at a power density of 0.8 W cm^-2^), the afterglow images of the tumors' largest cross-section were collected using the IVIS *in vivo* imaging system.

CDSP NPs (at a CDs/PTX ratio of 15/22.5 mg mL^-1^, 200 μL) were placed in 96-well plates and pre-irradiated with a 685 nm laser (at a power density of 0.8 W cm^-2^) for 5 min. Then, live Balb/c mice were placed over them. After the laser was removed, afterglow imaging was recorded in bioluminescence mode using an open filter.

**Afterglow imaging-guided resection of 4T1 tumor.**

Balb/c mice with a tumor size of approximately 100 mm^3^ were randomly divided into two groups. CDSP NPs (at a CDs/PTX ratio of 15/22.5 mg mL^-1^, 50 μL) were injected intratumorally into the Balb/c mice, and surgical resection was carried out 2 h later under the guidance of fluorescence or afterglow imaging. Preoperative fluorescence or afterglow imaging was performed to determine the tumor location and guide the first surgical resection. After the first resection, fluorescence or luminescence imaging was repeated to identify any tumor remnants for a second precise excision. Upon completion of the second resection, imaging was conducted on the mice to ensure that no luminescent signal was detected, signifying that all tumor tissue had been removed. At this stage, the wound was sutured and monitored regularly. The body weight and tumor volume of the mice were measured every 2 days. After 24 d following the surgical resection, the mice were sacrificed and the tumors were collected to evaluate the tumor recurrence rate.

***In vivo* photodynamic combined chemotherapy**

When the tumors reached a volume of approximately 80 mm^3^, the mice were randomly divided into seven groups (n = 5 per group) and treated with PBS, PBS + L, CDs, CDs + L, Abraxane, CDSP NPs, and CDSP NPs + L. Different reagents were intravenously injected into the mice at equivalent CDs/PTX doses of 10/15 mg/kg (drug weight/body weight) on day 1. Twelve hours after injection, the mice treated with PBS + L, CDs + L, and CDSP NPs + L were anesthetized, and the tumor sites were irradiated with a laser (685 nm, 0.3 W cm^-2^) for 10 min. The same treatment was repeated once on day 3. The body weight and tumor volume of the mice were measured every 2 d. On day 14, the mice were sacrificed and the tumors were removed to evaluate the tumor suppression efficacy. The major organs (heart, liver, spleen, lung, and kidney) and tumors were collected, fixed in 4% paraformaldehyde solution, paraffin-embedded, sectioned, and stained with hematoxylin and eosin (H&E) for histopathological analysis.

**Figure S1.** XRD pattern of CDs.

**Figure S2.** The excitation spectrum of CDs in ethanol solution.

**Figure S3.** Images of CDs in aqueous solution: (left) under daylight, (middle) fluorescence (λex = 400 nm, λem = 680 nm), and (right) afterglow images (CDs: 1 mg mL^-1^).

**Figure S4.** High-resolution O 1s XPS spectrum of CDs.

**Figure S5.** ESR signals of TEMP-singlet oxygen (^1^O_2_) spin adducts for the groups treated with CDs + L (685 nm laser, 0.1 W cm^-2^, 5 min), CDs + L + Vitamin C, and CDs.

**Figure S6.** The photograph of the CDSP-0.1 NPs, CDSP-0.3 NPs, CDSP-0.5 NPs, CDSP-1 (CDSP), CDSP-3 NPs and CDSP-10 NPs aqueous solution under sunlight (CDs: 100 μg mL^-1^).

**Figure S7.** Size changes of CDSP NPs treated with (a) PBS contained 10% FBS, (b) RPMI 1640, and (c) 5% glucose solution for 24 h. Bars represent SD (n = 3).

**Figure S8.** The afterglow spectra of CDs in ethanol solution and CDSP NPs aqueous solution by IVIS in bioluminescence mode.

**Figure S9.** Corresponding overlapped profiles of red fluorescence from CDSP NPs with green fluorescence from Lyso Tracker along the white selected line across the cell.

**Figure S10.** Flow cytometry analysis of ROS production in 4T1 cells treated with PBS, PBS + L, CDs, CDs + L, Abraxane, CDSP NPs, and CDSP NPs + L, followed by incubation with the DCFH-DA probe (CDs/PTX concentration: 10/15 μg mL^-1^, irradiated with 685 nm light at a power of 0.4 W cm^-2^ for 5 min).

**Figure S11.** Afterglow imaging of a 4T1 tumor-bearing mouse after intratumoral injection of CDSP NPs (CDs/PTX:15/22.5 mg mL^-1^, 50 μL). The tumor site was preirradiated with laser (685 nm, 0.8 W cm^-2^, 10 min), and then the images were collected after removing the laser.

**Figure S12.** (a) The afterglow imaging and (b) daylight photograph of a mouse placed on top of a pre-irradiated CDSP NPs solution.

**Figure S13.** Routine blood analysis of the mice after treatment with PBS, PBS + L, CDs, CDs + L, Abraxane, CDSP NPs, and CDSP NPs + L.

**Figure S14.** H&E-stained sections of main organs in PBS, PBS + L, CDs, CDs + L, Abraxane, CDSP NPs, and CDSP NPs + L groups.

References

[1] J. Wang, Q. Pei, R. Xia, S. Liu, X. Hu, Z. Xie, X. Jing, Comparison of Redox Responsiveness and Antitumor Capability of Paclitaxel Dimeric Nanoparticles with Different Linkers. *Chem. Mater.* **2020**, *32*, 10719.
